# Dietary Betaine Addition Promotes Hepatic Cholesterol Synthesis, Bile Acid Conversion, and Export in Rats

**DOI:** 10.3390/nu12051399

**Published:** 2020-05-13

**Authors:** Sisi Li, Shuyi Xu, Yang Zhao, Haichao Wang, Jie Feng

**Affiliations:** 1 Key Laboratory of Molecular Animal Nutrition, Ministry of Education, College of Animal Sciences, Zhejiang University, Hangzhou 310027, China; lisisi@zju.edu.cn (S.L.); ripple_xsy@zju.edu.cn (S.X.); 21817006@zju.edu.cn (Y.Z.); 2 Department of Animal Science, College of Life Sciences and Food Engineering, Hebei University of Engineering, Handan 430068, China; wanghaichao@hebeu.edu.cn

**Keywords:** betaine, cholesterol metabolism, cholesterol synthesis, cholesterol transport, bile acids efflux

## Abstract

It is widely reported how betaine addition regulates lipid metabolism but how betaine affects cholesterol metabolism is still unknown. This study aimed to investigate the role of betaine in hepatic cholesterol metabolism of Sprague-Dawley rats. Rats were randomly allocated to four groups and fed with a basal diet or a high-fat diet with or without 1% betaine. The experiment lasted 28 days. The results showed that dietary betaine supplementation reduced the feed intake of rats with final weight unchanged. Serum low-density-lipoprotein cholesterol was increased with the high-fat diet. The high-fat diet promoted cholesterol synthesis and excretion by enhancing the HMG-CoA reductase and ABCG5/G8, respectively, which lead to a balance of hepatic cholesterol. Rats in betaine groups showed a higher level of hepatic total cholesterol. Dietary betaine addition enhanced cholesterol synthesis as well as conversion of bile acid from cholesterol by increasing the levels of HMGCR and CYP7A1. The high-fat diet decreased the level of bile salt export pump, while dietary betaine addition inhibited this decrease and promoted bile acid efflux and increased total bile acid levels in the intestine. In summary, dietary betaine addition promoted hepatic cholesterol metabolism, including cholesterol synthesis, conversion of bile acids, and bile acid export.

## 1. Introduction

Betaine is a trimethyl derivative of the amino acid glycine. It is widely found in common food, including shellfish, flour, grains, and some vegetables [1,2,3]. Betaine in mammals is mainly absorbed from food and the synthesis of choline in vivo, while dietary betaine leads to a transient increase in tissue [4]. Betaine mainly functions as an osmotic regulator and methyl donor in the body. As an organic osmolyte, betaine accumulates in most tissues to assist with cell volume regulation [5,6] and improves intestinal function [7]. Due to its special molecular structure, betaine is also an efficient methyl donor. The transmethylation of betaine is used in many biochemical pathways including the methionine–homocysteine cycle and the biosynthesis of carnitine and phospholipids [8,9,10]. Thus, betaine plays an important role in lipid metabolism including decrease of hepatic triglyceride accumulation [11,12] and increase of intramuscular fat content [13,14]. Research from human studies also showed that betaine supplementation can reduce body fat, ameliorate nonalcoholic steatohepatitis, and prevent the deterioration of steatosis [15,16,17].

Cholesterol is one of the most important lipoids with a wide range of physiological functions in the body. The stable metabolism of cholesterol plays an irreplaceable role during healthy animal growth. The liver plays a central role in maintaining cholesterol balance in different animal species [18]. Hepatic cholesterol metabolism is involved in uptake, synthesis, efflux, and elimination [19]. All these processes are regulated by key factors or rate-limited enzymes. Dietary cholesterol is transported by low-density lipoprotein (LDL) and are taken into the liver by the LDL receptor (LDLR) [20]. Excess cholesterol in peripheral tissues is transported by high-density lipoprotein (HDL) to the liver and Scavenger Receptor Class B Type I (SRBI) mediates the cholesterol entering the hepatocyte from HDL [21]. Hepatocytes can synthesize cholesterol from acetyl-CoA catalyzed by 3-hydroxy-3-methyl glutaryl coenzyme A reductase (HMGCR) [22]. Conversion into bile acids is the major catabolic route for cholesterol disposal in the liver. Hepatic bile acid export is mediated by the ATP-binding cassette transporter bile salt export pump (BSEP) [21,23]. ATP binding cassette subfamily G member 5 (ABCG5) and member 8 (ABCG8) heterodimerize into a functional complex that is crucial for hepatobiliary cholesterol excretion [24].

Previous studies have reported that maternal betaine may affect progeny cholesterol metabolism through epigenetic regulation of related genes [25,26,27]. However, results from research of dietary betaine’s effect on cholesterol by the nutritional pathway have been inconsistent. Dietary betaine supplementation increases the concentration of total cholesterol in muscle and adipose tissue in pigs [28,29]. The plasma total cholesterol level was not changed by betaine addition in pigs and mice [30,31]. Others reported that plasma cholesterol was increased in rats and decreased in mice fed with long-term betaine supplementation [32]. Research involving humans showed that betaine was negatively correlated with non-HDL cholesterol [33,34]. In general, dietary betaine may affect the levels of cholesterol in plasm and different tissues of animal bodies. The specific mechanism by which betaine regulates cholesterol metabolism requires further research. Thus, the present study aimed to investigate the effects of dietary betaine supplementation on liver cholesterol metabolism in Sprague-Dawley (SD) rats fed with either a normal or high-fat diet to determine how betaine regulates the key factors involved in hepatic cholesterol metabolism.

## 2. Materials and Methods

### 2.1. Animals and Experimental Design

The experimental protocol used in this research was approved by the Institutional Animal Ethics Committee of Zhejiang University. The experiment was carried out in the Laboratory Animal Center of Zhejiang University, Hangzhou, China. All experimental procedures were undertaken with reference to the Guidelines for the Care and Use of Laboratory Animals in China. A total of 32 6-week-old female SD rats (Laboratory Animal Center of Zhejiang Academy of Medical Sciences, Hangzhou, China), weighing 186.42 ± 6.16 g, were randomly allocated to four groups. Animals were fed with different diets: basal diet (C), basal diet supplemented with 1% betaine (CB), high-fat diet (HF), high-fat diet supplemented with 1% betaine (HFB). The betaine used in this study was provided by Healthy Husbandry Sci-tech Co., Ltd. Hangzhou, China. The energy composition of the basal diet included 13.8% derived from fat, 25.7% from protein, and 60.5% from carbohydrates, while for the high-fat diet this was 40.0% from fat, 20.0% from protein, and 40.0% from carbohydrates. The cholesterol content in the basal diet was less than 0.2 mg/kg. The high-fat diet contained 0.017% cholesterol. The diets were formulated in accordance with AIN-93G (American Institute of Nutrition, Rockville, MD, USA [35]) and the formula is shown in Table 1. All diets were specially provided by SLAC Experimental Animals Co., Ltd. (Shanghai, China). The rats were housed with a schedule of 12 h light and 12 h darkness with constant temperature (21 ± 1 °C) and humidity (75% ± 5%). Animals were individually caged in stainless steel with free access to a chow diet and water throughout the entire feeding period. The body weight of each rat and feed intake was recorded once a week. The experiment lasted for 28 days.

### 2.2. Sample Collection

At the end of the trial, the rats were anesthetized with chloral hydrate. The serum samples were collected from orbital blood and then centrifuged at 3000× *g* for 10 min at 4 °C and stored at −80 °C. The rats were then euthanized by cervical dislocation, and fresh livers were weighed and collected. A small piece of fresh liver was fixed in 4% paraformaldehyde for oil red O staining. The remainder was frozen in liquid nitrogen immediately and stored at −80 °C for subsequent analysis.

### 2.3. Analysis of Lipid Metabolites in Serum

The levels of total triglyceride (TG), total cholesterol (TC), high-density-lipoprotein cholesterol (HDLC), low-density-lipoprotein cholesterol (LDLC), and total bile acid (TBA) in serum were measured by analysis kits (A110-2, A111-2, A112-1, A113-1, and E003-2-1, Jiancheng Institute of Biotechnology, Nanjing, China). Non-esterified fatty acid (NEFA) was measured by an automatic biochemical analyzer (Olympus Au5400). The concentrations of very-low-density-lipoprotein-cholesterol (VLDL-C) and lysophosphatidylcholine (LPC) were measured by enzyme-linked immunosorbent assay kits (H249, Jiancheng, Nanjing and CEK621Ge, Cloud-clone corp, Wuhan, China, respectively) according to the instruction manual.

### 2.4. Hepatic Histology and Metabolites Analysis

The specimens of liver were fixed in 4% paraformaldehyde for 24 h and then stained with oil red O as previously described [12]. The level of total cholesterol and total triglyceride in liver of rats was measured by kits (E1015 and E1013, Applygen, Beijing, China). A 10% hepatic homogenate was prepared with the lysis buffer provided in the kits before measurement according to the operation manual. The levels of acetyl coenzyme A (Ac-CoA) and carnitine palmitoyl transferase 1 (CPT1) were measured by enzyme-linked immunosorbent assay kits (H230, Jiancheng Institute of Biotechnology, Nanjing, China). A BCA-kit (P1511, Applygen, Beijing, China) was used to measure the protein concentration in the liver. The levels of hepatic and intestinal TBA were measured by commercial kits from Jiancheng bioengineering Institute (Nanjing, China) according to the operating instructions.

### 2.5. Western Blot Analysis

Protein from liver samples was extracted by RIPA Lysis Buffer (P0013, beyotime, Shanghai, China) containing 1 mmol/L protease inhibitor (ST506, PMSF, beyotime, Shanghai, China) and quantified with a BCA protein assay kit (KGP902, keygentec, Nanjing, China) according to kit instructions. Proteins were separated on SDS-PAGE and then electrophoretically transferred onto PVDF membrane (Millipore, Code No. IPVH00010, Burlington, MA, USA). Membranes were blocked for 2 h in TBST containing 5% nonfat dry milk at room temperature. Then, membranes were incubated overnight at 4 °C in antibody dilution buffer containing primary antibodies (details are shown in Table 2). A goat anti-rabbit IgG (H + L) secondary antibody (Bioker biotechnology, code BK-M050, Hangzhou, China) with 1/20,000 dilution was used in the detection of specific proteins. GAPDH was used as control. Finally, the signals were detected by adding ECL Star Chemiluminescence solution (P0018, Beyotime Biotechnology, Shanghai, China). Band intensities were determined by using Image J software. The relative expression of target proteins = the optical density of target proteins/the optical density of GAPDH.

### 2.6. Treatments of Cells

HepG2 and BRL3A cell lines were all from American Type Culture Collection (ATCC, Manassas, VA, USA). Human hepatocellular carcinoma cell HepG2 and rat liver fibroblast BRL3A were cultured in DMEM added with 10% FBS, penicillin (100 U/mL), and streptomycin (100 μg/mL). All cell lines were grown in humidified tissue culture incubator (SANYO) with an atmosphere of 5% CO_2_ at 37 °C. The method concerning a high-fat medium reported by Lee et al. [36] was preferred. The free fatty acid (FFA) mixture (oleic acid/palmitic acid, 1:1) was prepared with fat-free bovine serum albumin (BSA, B2064, sigma, St. Louis, MO, USA). After attaining 70% confluence, cells were cultured with normal medium or exposed to medium containing 1 mmol/L FFA, with or without 10 mmol/L betaine, for 24 h. The level of TC in cells was measured by a cholesterol cell-based detection assay kit (No. 10009779, Cayman, Ann Arbor, MI, USA). The level of cholesterol uptake was determined by a cholesterol uptake cell-based assay kit (No. 600440, Cayman, Ann Arbor, MI, USA) according to the operation manual.

### 2.7. Statistical Analysis

Results are presented as means and standard deviations. Data analyses were performed with the SPSS 25.0 statistical software (IBM). Statistical analysis was performed by two-way ANOVA, and the simple effect analysis of the LSD method was used for post-test analysis. In all analyses, the level of significant difference was set at *p* < 0.05.

## 3. Results

### 3.1. Growth Performance

**Figure 1:**
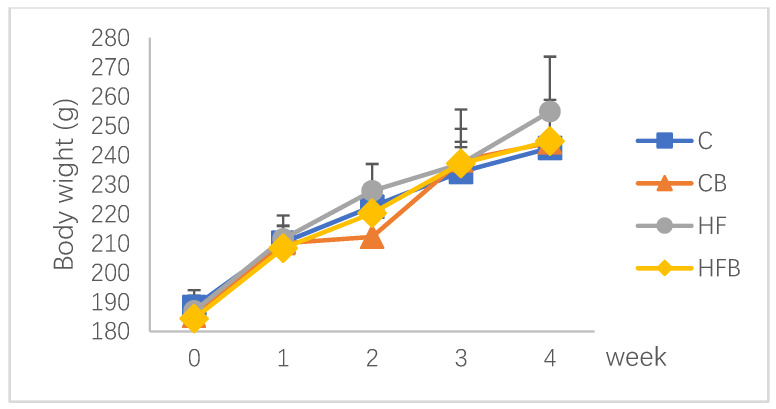
Effects of betaine and high-fat diet on body weight growing on rats (*n* = 8). C—basal diet, CB—basal diet supplemented with 1% betaine, HF—high-fat diet, HFB—high-fat diet supplemented with 1% betaine.

Figure 1 shows the body weight increase of rats during the trial period. There was no significant difference among the groups (*p* > 0.05). The final body weight of rats was not affected by dietary betaine addition nor high-fat diet (Table 3, *p* > 0.05). In addition, rats in the high-fat group showed a numerically higher body weight than other groups.

**Figure 2:**
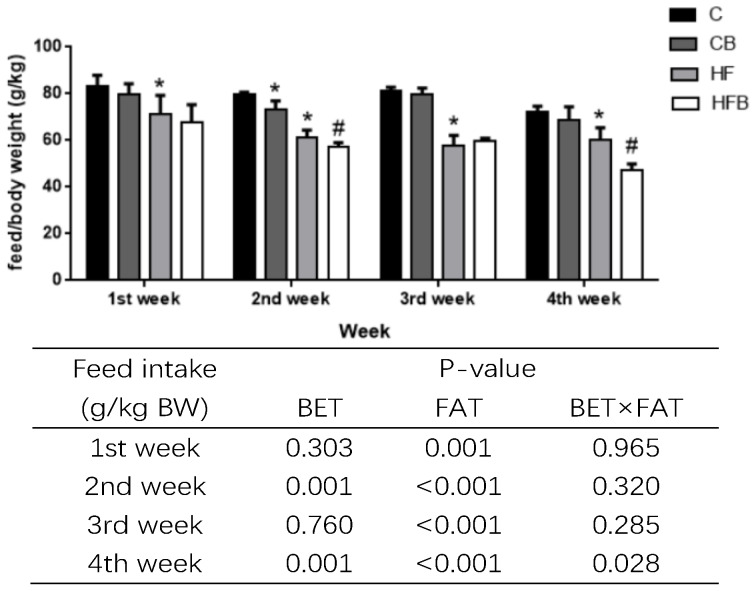
Effects of betaine and high-fat diet on daily feed intake of rats during the trial period (*n* = 8). C—basal diet, CB—basal diet supplemented with 1% betaine, HF—high-fat diet, HFB—high-fat diet supplemented with 1% betaine. * Significantly different from the C group (*p* < 0.05). # Significantly different from the HF group (*p* < 0.05).

As shown in Figure 2, the high-fat diet remarkably reduced feed intake of rats during the trial period including the feed intake in each week and average feed intake throughout the whole period (*p* < 0.05). Dietary betaine addition significantly decreased the feed intake of rats in the second and fourth week, and the average feed intake throughout the whole period was also reduced by betaine addition (*p* < 0.05). Additionally, rats supplemented with betaine showed lower average feed intake when fed with a high-fat diet (*p* < 0.05). The average daily weight gain of rats was significantly increased by the high-fat diet (Table 3, *p* < 0.05). The index of liver was not affected by high-fat nor betaine in rats (*p* > 0.05).

### 3.2. Serum Lipid Metabolites

Table 4 shows the effects of betaine and high-fat diet on serum lipid metabolites of rats. The concentration of serum TG was not affected by betaine addition nor high-fat diet (*p* > 0.05). The level of serum NEFA was reduced by betaine addition (*p* < 0.05) rather than the high-fat diet. Betaine addition increased the concentration of serum VLDL-C (*p* < 0.05), which was not affected by high-fat diet. There was a significant difference in the level of LPC affected by the interaction of betaine and high-fat diet (*p* < 0.05). Betaine addition also showed a significant effect on the LPC level (*p* < 0.05). Rats in the CB group showed a higher level of LPC than the control group. The level of TC and HDLC showed no significant difference among groups (*p* > 0.05). The two-way ANOVA showed that the level of LDLC was significantly affected by the high-fat diet (*p* < 0.05). The level of serum TBA was decreased by the high-fat diet (*p* < 0.05). Rats fed with betaine addition showed a lower TBA level compared to the control group (*p* < 0.05). However, the influence of betaine on serum TBA level was not significant (*p* > 0.05).

### 3.3. Hepatic Metabolite

**Figure 3:**
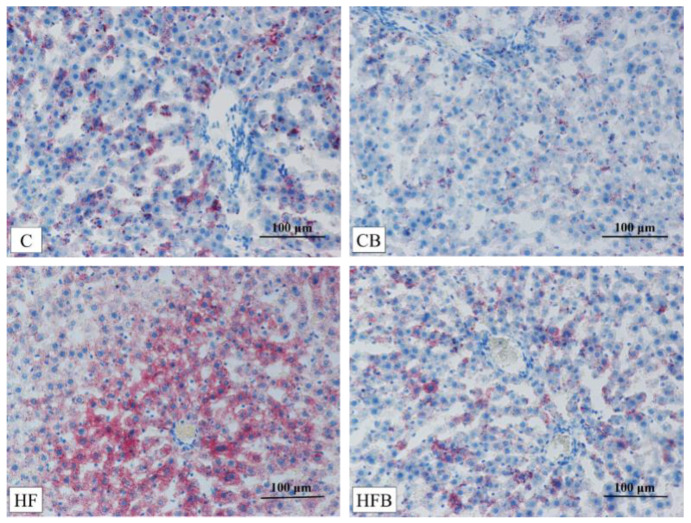
The results of betaine and high-fat diet on lipid deposition in liver of rats (200×). C—basal diet, CB—basal diet supplemented with 1% betaine, HF—high fat diet, HFB—high fat diet supplemented with 1% betaine.

The dye of oil red O can bind to neutral lipids to show small lipid droplets and dye it with a red color. The results of liver staining with oil red O are shown in Figure 3. The high-fat diet promoted lipid accumulation in the liver of rats (fat droplets were dyed with red), while betaine supplementation prevented lipid-accumulation, especially in rats fed with a high-fat diet.

**Figure 4:**
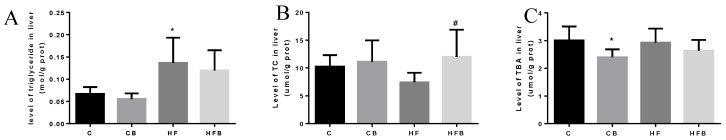
Effects of betaine and high-fat diet on hepatic metabolites in rats (*n* = 8). * Significantly different from the C group (*p* < 0.05). # Significantly different from the HF group (*p* < 0.05). (**A**) Level of hepatic triglyceride. (BET, *p* = 0.303, FAT, *p* < 0.001, interaction, *p* = 0.836). (**B**) Level of hepatic total cholesterol (TC) (BET, *p* = 0.036, FAT, *p* = 0.436, interaction, *p* = 0.145). (**C**) Level of total bile acids (TBA) in the liver (BET, *p* = 0.006, FAT, *p* = 0.604, interaction, *p* = 0.314).

As shown in Figure 4, the concentration of hepatic triglyceride in rats was remarkably increased by high-fat diet (*p* < 0.05). Betaine addition reduced the level of hepatic triglyceride by 16.8% and 12.3% compared to the control group and high-fat-diet group, respectively, although these changes were not significant. The level of hepatic TC and TBA in rats was not affected by a high-fat diet (*p* > 0.05). The hepatic total cholesterol concentration was significantly affected by betaine addition (*p* < 0.05). Dietary betaine addition increased the level of TC in the liver when rats were fed with a high-fat diet. There was a significant effect of betaine on the level of TBA in the liver (*p* < 0.05). Rats fed with betaine addition showed a lower hepatic TBA level compared to the control group.

### 3.4. Key Factors Involved in Cholesterol Metabolism

#### 3.4.1. Cholesterol Synthesis

**Figure 5:**
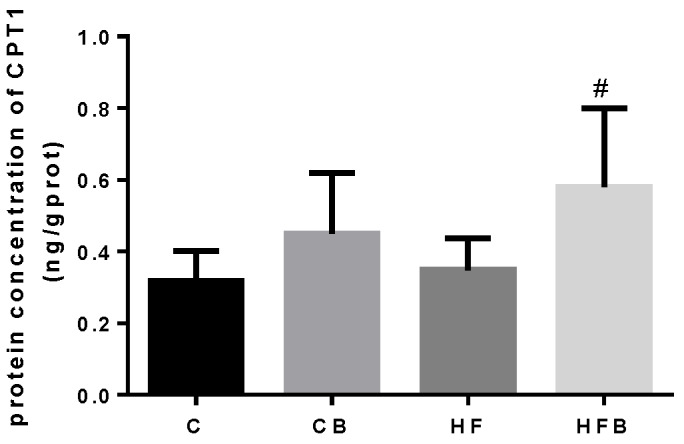
Effects of betaine and high-fat diet on concentration of CPT1 in the liver of rats (*n* = 8): the concentration of hepatic Carnitine palmitoyl transferase1 (CPT1, BET, *p* = 0.002, FAT, *p* = 0.148, interaction, *p* = 0.361). # Significantly different from the HF group (*p* < 0.05).

**Figure 6:**
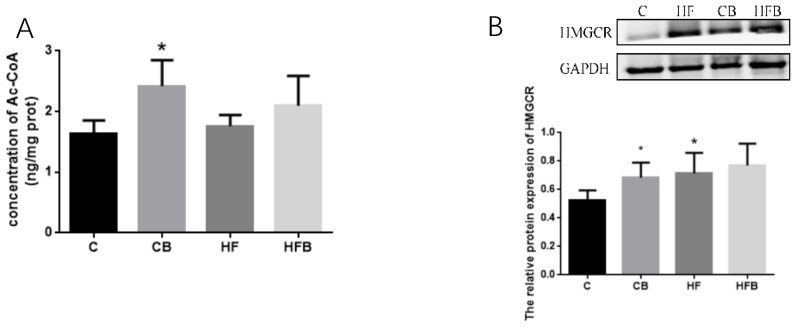
Effects of betaine and high-fat diet on hepatic cholesterol synthesis of rats (*n* = 8). (**A**) Concentration of hepatic acetyl coenzyme A (Ac-CoA, BET, *p* < 0.001, FAT, *p* = 0.450, interaction, *p* = 0.083). (**B**) HMGCR: HMG CoA reductase (BET, *p* = 0.073, FAT, *p* = 0.025, interaction, *p* = 0.359). * Significantly different from the C group (*p* < 0.05). C—basal diet, CB—basal diet supplemented with 1% betaine, HF—high-fat diet, HFB—high-fat diet supplemented with 1% betaine.

CPT1 is a key enzyme for β-oxidization of fatty acid in liver. Ac-CoA is a vital material for cholesterol biosynthesis. As shown in Figure 5 and Figure 6, the levels of hepatic CPT1 and AC-CoA were both significantly increased by betaine addition rather than affected by the high-fat diet in rats (*p* < 0.05). HMGCR is the rate-limiting enzyme during cholesterol synthesis in liver. As can be seen from Figure 6, the high-fat diet remarkably enhanced the level of hepatic HMGCR in rats (*p* < 0.05). Furthermore, betaine addition increased the level of hepatic HMGCR in rats compared to the control group (*p* = 0.05), but its addition showed no effect on the hepatic HMGCR level when rats were fed with a high-fat diet (*p* > 0.05). A two-way ANOVA test showed that betaine’s effect on the hepatic HMGCR level was not significant (*p* > 0.05).

#### 3.4.2. Cholesterol Transport

**Figure 7:**
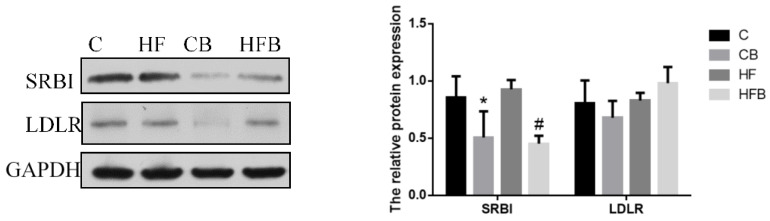
Effects of betaine and high-fat diet on protein expression of hepatic LDLR and SRBI in rats (*n* = 8). SRBI, class B scavenger receptor (BET, *p* < 0.001, FAT, *p* = 0.918, interaction, *p* = 0.475). LDLR, low-density lipoprotein receptor (BET, *p* = 0.839, FAT, *p* = 0.011, interaction, *p* = 0.027). * Significantly different from the C group (*p* < 0.05). # Significantly different from the HF group (*p* < 0.05). C—basal diet, CB—basal diet supplemented with 1% betaine, HF—high-fat diet, HFB—high-fat diet supplemented with 1% betaine.

LDLR and SRBI are cell surface receptors in hepatocytes mediating the uptake of LDLC and HDLC, respectively. The level of hepatic LDLR was significantly affected by a high-fat diet (*p* < 0.05, Figure 7). Although betaine addition showed no influence on the hepatic LDLR level, the effect of the interaction (betaine and high-fat diet) was significant (*p* < 0.05). As shown in Figure 7, dietary betaine supplementation significantly reduced the level of hepatic SRBI (*p* < 0.05) which was not affected by a high-fat diet.

#### 3.4.3. Cholesterol Elimination

**Figure 8:**
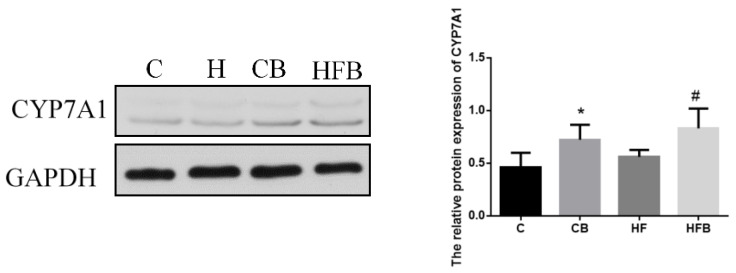
Effects of betaine and high-fat diet on the level of hepatic CYP7A1 of rats (*n* = 8). CYP7A1, Cholesterol 7α-hydroxylase (BET, *p* = 0.007, FAT, *p* = 0.220, interaction, *p* = 0.943). * Significantly different from the C group (*p* < 0.05). # Significantly different from the HF group (*p* < 0.05). C—basal diet, CB—basal diet supplemented with 1% betaine, HF—high-fat diet, HFB—high-fat diet supplemented with 1% betaine.

Converting bile acids in the liver is one of the most important ways to eliminate cholesterol from the body. Cholesterol 7α-hydroxylase (CYP7A1) was the key enzyme during bile acid synthesis from cholesterol. Immunoblot analysis revealed that the protein level of CYP7A1 was significantly increased by dietary betaine supplementation (*p* < 0.05, Figure 8). The high-fat diet showed no effect on CYP7A1 level (*p* > 0.05).

**Figure 9:**
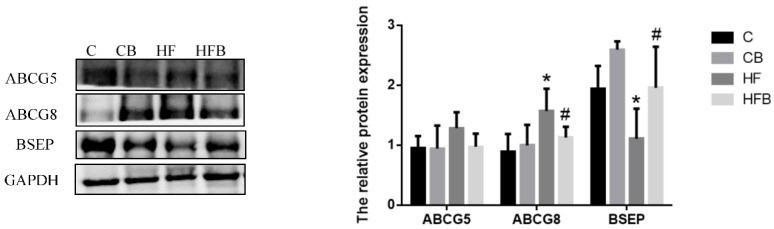
Effects of betaine and high-fat diet on factors involving cholesterol excretion in liver of rats (*n* = 8). ABCG5, ATP Binding Cassette Subfamily G Member 5 (BET, *p* = 0.297, FAT, *p* = 0.242, interaction, *p* = 0.327). ABCG8, ATP Binding Cassette Subfamily G Member 8 (BET, *p* = 0.068, FAT, *p* = 0.011, interaction, *p* = 0.242). BSEP, Bile salt export pump (BET, *p* = 0.013, FAT, *p* = 0.014, interaction, *p* = 0.704). * Significantly different from the C group (*p* < 0.05). # Significantly different from the HF group (*p* < 0.05). C—basal diet, CB—basal diet supplemented with 1% betaine, HF—high-fat diet, HFB—high-fat diet supplemented with 1% betaine.

ABCG5 and ABCG8 are ATP-binding cassette transporters that promote biliary excretion of neutral sterols. The protein level of hepatic ABCG8 was significantly increased by a high-fat diet (*p* < 0.05, Figure 9). Betaine addition reduced the level of ABCG8, which was increased in the high-fat diet, but a two-way ANOVA test did not show a significant difference in betaine affect. The effects of betaine addition and high-fat diet on hepatic ABCG5 had similar tendencies to ABCG8 but did not reach a significant difference (Figure 9, *p* > 0.05). The bile salt export pump (BSEP) constitutes the rate limiting step of the hepatocellular bile salt transport. As shown in Figure 9, the high-fat diet reduced the level of hepatic BSEP in rats (*p* < 0.05). Dietary betaine addition significantly affected the level of hepatic BSEP (*p* < 0.05). In particular, when rats were fed with a high-fat diet, betaine remarkably increased the BSEP level in the liver.

**Figure 10:**
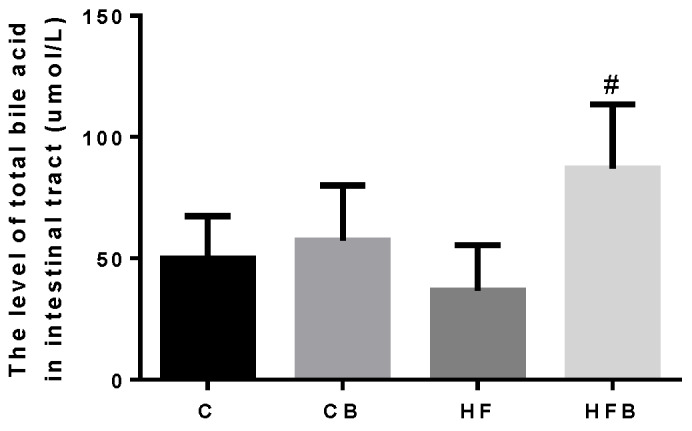
Effects of betaine and high-fat diet on total bile acid in intestine of rats (*n* = 8). Two-way ANOVA: BET, *p* = 0.041, FAT, *p* = 0.211, interaction, *p* = 0.143. C—basal diet, CB—basal diet supplemented with 1% betaine, HF—high-fat diet, HFB—high-fat diet supplemented with 1% betaine. # Significantly different from the HF group (*p* < 0.05).

Figure 10 shows the level of TBA in the intestine of rats. Betaine addition significantly increased the TBA level in the intestine (*p* < 0.05). In particular, in rats fed with a high-fat diet, the supplementation of betaine showed a significant higher intestinal TBA level than the high-fat group (*p* < 0.05).

### 3.5. Cholesterol of Hepatocyte In Vitro

**Figure 11:**
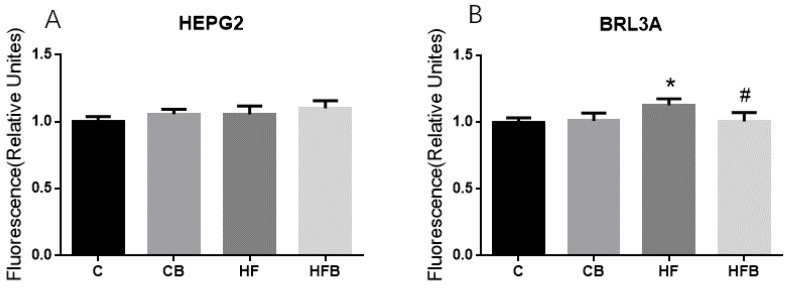
Effects of betaine and high-fat-medium on the cholesterol level of hepatic cells in vitro (*n* = 6). (**A**) The level of total cholesterol in HepG2: BET, *p* = 0.019, FAT, *p* = 0.022, interaction, *p* = 0.763. (**B**) The level of total cholesterol in BRL3A: BET, *p* = 0.017, FAT, *p* = 0.008, interaction, *p* = 0.006. C—normal medium; CB—normal medium supplemented with 10 mmol/L betaine; HF—high-fat-medium containing 1 mmol/L FFA; HFB—medium containing 1 mmol/L FFA with 10 mmol/L betaine. * Significantly different from the C group (*p* < 0.05). # Significantly different from the HF group (*p* < 0.05).

In order to further investigate the effects of betaine addition on cholesterol metabolism in hepatocytes, we carried out the experiment on hepatocytes in vitro. As shown in Figure 11, there was a significant difference of cholesterol level in HepG2 cells affected by betaine or high-fat medium (*p* < 0.05). As for BRL3A cells, betaine addition or high-fat medium also showed a significant effect on the cholesterol level (*p* < 0.05). Additionally, betaine addition decreased the level of cholesterol in BRL3A, which was increased by the high-fat medium.

**Figure 12:**
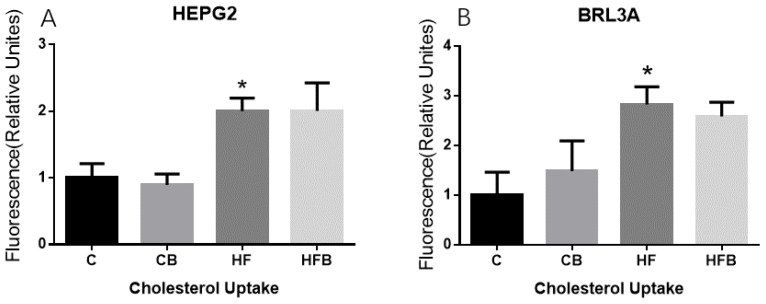
Effects of betaine and high-fat medium on cholesterol uptake of hepatic cells in vitro (*n* = 6). (**A**) The level of cholesterol uptake in HepG2: BET, *p* = 0.647, FAT, *p* < 0.001, interaction, *p* = 0.615. (**B**) The level of cholesterol uptake in BRL3A: BET, *p* = 0.546, FAT, *p* < 0.001, interaction, *p* = 0.090. C—normal medium, CB—normal medium supplemented with 10 mmol/L betaine; HF—high-fat-medium containing 1 mmol/L FFA; HFB—medium containing 1 mmol/L FFA with 10 mmol/L betaine. * Significantly different from the C group (*p* < 0.05).

NBD cholesterol, a fluorescently-tagged cholesterol, was supplemented in the medium as a probe for the detection of cholesterol uptake in hepatocytes. The results suggested that cholesterol uptake of HepG2 and BRL3A was significantly enhanced with a high-fat medium (Figure 12, *p* < 0.001), while betaine addition had no influence on cholesterol uptake of these hepatic cells (Figure 12, *p* > 0.05).

## 4. Discussion

This study investigated the effects of betaine addition on growth performance in SD rats. Many studies have investigated betaine’s effect on growth performance in animals and the results are variable [37]. In this study, betaine supplementation resulted in a decreased average daily intake without the final body weight being affected. It might reveal that the feed conversion efficiency was improved by betaine addition, since betaine addition could increase the digestibility of protein and fat, as previously reported [2]. This finding is consistent with most previous research on betaine [38]. In this study, it may be related to betaine promoting the efflux of bile acid, thereby increasing the digestion and absorption of fat in a high-fat diet. With the basal diet, the effect of betaine increasing the digestion and absorption of fat was not as significant as it was in the high-fat diet. The present results also found that betaine addition increased the concentration of serum VLDL and LPC, which involved lipid transport in the circulation system of rats. All these results indicated that betaine addition may improve the utilization of feed energy [39].

This study also investigated the effects of betaine supplementation on cholesterol metabolism in SD rats. Liver plays a central role in maintaining cholesterol balance [18]. It is in the liver rather than in the extrahepatic tissues that there would be changes to accommodate any alteration in net sterol balance in an animal [40]. After four weeks of high-fat-diet feeding, the level of hepatic triglyceride was increased without changing the hepatic total cholesterol concentration in rats. However, the protein level of hepatic HMGCR, the rate-limiting enzyme in synthesis of cholesterol [22], was increased in this study. It was reported that a high-fat diet could enhance the activity of hepatic HMGCR with hepatic total cholesterol unchanged in mice [41]. Previous studies in pigs found that long-term betaine feeding increased the level of cholesterol in adipose tissue and muscle tissue [29,30]. Maternal dietary betaine supplementation also increased the concentration of hepatic total cholesterol in pigs and chicks [26,28]. Results of the present study showed that betaine addition significantly increased the level of total cholesterol in the liver of rats and in hepatocytes in vitro. The protein level of hepatic HMGCR in rats was increased by betaine addition with hepatic CPT1 and Ac-CoA levels increased in this study. Ac-CoA was both a production of fatty acid oxidation and a key substrate in the biosynthesis of cholesterol [42]. CPT1 was a key rate-limiting enzyme of fatty acid β-oxidation [43]. The experiment in vitro showed that the increase of cholesterol levels in hepatocytes by betaine supplementation was not due to cholesterol uptake. Thus, dietary betaine may promote the de novo biosynthesis of cholesterol from acetyl-CoA by increasing the level of HMGCR. Interestingly, we found that betaine addition reduced the cholesterol level in BRL3A cells, which was increased by the high-fat medium. However, when the level of hepatic cholesterol was not affected by a high-fat diet, betaine addition increased the cholesterol concentration with high-fat diet feeding. Other studies reported no effect of betaine on the hepatic cholesterol level with a normal diet nor a high-fat diet [12,32]. Nevertheless, liver cholesterol increased markedly by high-fat ethanol, which was significantly blunted in the betaine addition groups [44,45]. We speculate that the hepatic cholesterol level is strictly regulated in the body, and betaine addition to some extent may regulate liver cholesterol metabolism, although this requires further research to examine.

Mechanisms involved in cholesterol regulation include its synthesis, uptake, storage, and efflux [19]. The transport of cholesterol in plasma was accomplished by lipoproteins. LDL carries cholesterol and other lipids from the liver to tissues throughout the body. Since the high-fat diet included lard, rats from high-fat groups showed a higher level of serum LDLC in this study. Other studies have reported that plasma LDLC levels could be markedly elevated by feeding on increased amounts of triacylglycerol [43]. LDLR is a cell surface receptor that mediates the uptake of LDLC into hepatocyte [20]. Therefore, the hepatic LDLR protein level was associated with plasma LDLC concentrations [46]. With the increase of the serum LDLC level in high-fat-diet-feeding rats, the hepatic LDLR protein level was also affected by the interaction between betaine and the high-fat diet in this study. Rats in the high-fat group showed a higher level of LDLR compared to the control group with betaine supplemented. Previous research reported that maternal dietary betaine supplementation upregulated the protein content of LDLR in rats and chickens and increased the gene expression of LDLR in piglets [26,47,48]. However, whether betaine could regulate serum LDLC levels increased by the high-fat diet via enhancing LDLR needs further research.

Excess cholesterol in peripheral tissues must be transported to the liver by HDL for excretion [49]. This multi-step process is called reverse cholesterol transport [50]. SRBI mediated the uptake of HDLC into hepatocytes [21]. Hepatic SRBI overexpression was accompanied by decreased plasma HDLC levels [51], but rats were highly resistant to atherogenesis and, with high plasma HDL, this meant the level of HDLC in rats was hardly affected [52]. Our results show that the protein expression of SRBI was reduced by betaine supplementation and the level of serum HDLC was not affected. Results of our previous study showed that serum HDLC was remarkably reduced by dietary betaine addition in pigs [53]. Other researchers mentioned that serum HDLC could be increased or unaffected by betaine addition in pigs or mice [54,55]. HDL particles acted as a pivotal player in the reverse transport of cholesterol from peripheral tissues into the liver, and SRBI was a key transport protein. It revealed that dietary betaine addition showed an effect on endogenous cholesterol transport, but more research is required to illuminate the specific mechanism.

The elimination of cholesterol in the body includes conversion to bile acids in the liver and the secretion of cholesterol with bile acids into the biliary tract. ABCG5/G8, a heterodimeric transporter complex, was the main player in biliary cholesterol secretion. Hepatic overexpression of ABCG5/G8 enhances hepatobiliary secretion of cholesterol [56]. This study found that a high-fat diet increases the level of hepatic ABCG5/G8, which leads to the balance of total cholesterol in the liver of rats. Conversion of hepatic cholesterol to bile acids is the major pathway to eliminate excess cholesterol in the body. This step was catalyzed by Cholesterol 7α-hydroxylase (CYP7A1), the main rate-limiting enzyme of the classical bile acid synthesis pathway [57,58]. This study showed that betaine addition increased the level of CYP7A1 when the hepatic TBA concentration decreased. The reduced TBA concentration may be caused by the increased level of bile salt export pump (BSEP), which was primarily responsible for the secretion of bile acids in liver. Betaine was found to produce marked bile secretion in rats and rabbits [59]. In this study, high-fat diet inhibited the expression of BSEP in liver, which may tend to lead to cholestasis [60]. The results of the intestinal TBA level showed it was highly increased by betaine addition. Thus, the addition of betaine promoted the efflux of bile acid from the liver into the intestine and increased the level of TBA in intestine especially in rats fed with a high-fat diet. After being exported into the small intestine, bile acids promote the absorption of dietary lipids [61]. It was according to the result that the average feed intake of rats fed with a high-fat diet was reduced by betaine addition. This may be a new explanation for dietary betaine addition improving fat digestibility and enhancing energy utilization.

## 5. Conclusions

In conclusion, dietary betaine addition promoted hepatic cholesterol metabolism through cholesterol synthesis, conversion of bile acids, and bile acid export. Dietary betaine addition enhanced bile acid efflux by improving the level of the bile acid salts export pump, which was inhibited by a high-fat diet.

## Figures and Tables

**Table 1:** The nutrition formula of diet *.

Ingredient	Basal Diet (g/kg)	High-Fat Diet (g/kg)
Maize starch	504.5	293.24
Casein	230.48	227.58
Sucrose	100.00	194.60
Soybean oil	60.00	32.76
Lard oil	0.0	169.00
Fiber	50.00	27.30
Mineral Mix#	35.00	35.00
Vitamin Mix#	10.00	10.00
L-Cys	3.00	3.00
Choline bitartrate	2.50	2.50
Antioxidant	0.02	0.02

* The data of nutrient levels are analyzed values. # Vitamin mix and mineral mix are in accordance with the American Institute of Nutrition-93 guidelines.

**Table 2:** The primary antibody of Western blot.

Primary Antibody	Order Numbers	Dilution	Size, kDa
Anti-HMGCR	ab174830, Abcam	1/5000	97
Anti-SRBI	ab217318, Abcam	1/2000	60–82
Anti-LDLR	10785-1-AP, Proteintech	1/2500	150–160
Anti-CYP7A1	bs-21429R, bioss	1/1500	55
Anti-ABCB11/BSEP	orb259591, Biorbyt	1/3000	146
Anti-ABCG5	27722-1-AP, Proteintech	1/1500	68–72
Anti-ABCG8	orb228808, Biorbyt	1/1500	76

**Table 3:** Effects of betaine on growth performance of high-fat-diet-fed SD rats (*n* = 8).

	C	CB	HF	HFB	*p*-Value		
					BET	FAT	BET × FAT
Initial body weight, g	188.4 ± 3.9	185.3 ± 6.1	186.4 ± 7.2	184.9 ± 4.7	0.252	0.569	0.700
Final body weight, g	242.5 ± 10.0	244.4 ± 7.9	254.9 ± 29.7	244.9 ± 14.1	0.520	0.310	0.349
Average daily gain, g/d	1.93 ± 0.34	2.11 ± 0.27	2.46 ± 0.43 *	2.25 ± 0.32	0.598	0.035	0.060
Average daily feed intake, g/kg BW	79.12 ± 12	75.47 ± 6.09	62.84 ± 7.46 *	58.13 ± 8.37#	0.008	<0.001	0.743
Liver index, %	3.90 ± 0.22	3.84 ± 0.26	3.89 ± 0.57	3.80 ± 0.17	0.542	0.863	0.894

* Significantly different from the C group (*p* < 0.05). # Significantly different from the HF group (*p* < 0.05). C—basal diet, CB—basal diet supplemented with 1% betaine, HF—high-fat diet, HFB—high-fat diet supplemented with 1% betaine.

**Table 4:** Effects of betaine and high-fat diet on serum lipid metabolites of rats (*n* = 8).

	C	CB	HF	HFB	*p*-Value		
					FAT	BET	FAT × BET
TG, mmol/L	1.47 ± 0.43	1.50 ± 0.21	1.76 ± 0.60	1.77 ± 0.38	0.075	0.862	0.951
NEFA, mmol/L	0.66 ± 0.15	0.52 ± 0.11 *	0.70 ± 0.18	0.57 ± 0.55	0.435	0.008	0.932
VLDL-C, umol/mL	0.40 ± 0.11	0.53 ± 0.20	0.33 ± 0.17	0.53 ± 0.17 #	0.639	0.021	0.596
LPC, ug/mL	0.71 ± 0.09	1.15 ± 0.12 *	1.11 ± 0.09	1.10 ± 0.43	0.080	0.034	0.029
TC, mmol/L	2.19 ± 0.60	2.21 ± 0.51	2.38 ± 0.45	2.79 ± 0.77	0.080	0.331	0.370
HDLC, mmol/L	0.83 ± 0.20	0.93 ± 0.32	0.84 ± 0.15	0.92 ± 0.20	0.970	0.307	0.867
LDLC, mmol/L	0.38 ± 0.07	0.36 ± 0.06	0.42 ± 0.07	0.44 ± 0.12	0.045	0.845	0.538
TBA, umol/L	43.0 ± 19.5	16.0 ± 2.9 *	14.9 ± 6.7 *	25.0 ± 10.3	0.039	0.066	<0.001

* Significantly different from the C group (*p* < 0.05). # Significantly different from the HF group (*p* < 0.05). TG, triglyceride; NEFA, non-esterified fatty acid; VLDL-C, very-low-density-lipoprotein-cholesterol; LPC, lysophosphatidylcholine; TC, total cholesterol; HDLC, high-density lipoprotein cholesterol; LDLC, low-density lipoprotein cholesterol; TBA, total bile acid. C—basal diet, CB—basal diet supplemented with 1% betaine, HF—high-fat diet, HFB—high-fat diet supplemented with 1% betaine.

## Abbreviations

ABCG5, ATP Binding Cassette Subfamily G Member 5; ABCG8, ATP Binding Cassette Subfamily G Member 8; AC-CoA, acetyl coenzyme A; BSEP, Bile salt export pump; CPT1, Carnitine palmitoyl transferase1; CYP7A1, Cholesterol 7α-hydroxylase; HDL, high-density lipoproteins; HDLC, high-density lipoprotein cholesterol; HMGCR, 3-hydroxy-3-methyl-glutaryl-coenzyme A reductase (HMG CoA reductase); LDL, low-density lipoproteins; LDLC, low-density lipoprotein cholesterol; LDLR, low-density lipoprotein receptor; LPC, lysophosphatidylcholine; NEFA, non-esterified fatty acid; SRBI, Class B scavenger receptor; TBA, total bile acid; TC, total cholesterol; TG, triglyceride; VLDL, very-low-density-lipoprotein.
